# The Silence of the Nurse: One Suggested Reason

**Published:** 1918-08-17

**Authors:** 


*To the Editor of* The Hospital.

### THE SILENCE OF THE NURSE.

### One Suggested Reason.

*To the Editor of* The Hospital.

Sir,—The vivid yet essentially practical articles by "Ierne" interest me very much. She lays her finger on weak spots so unnerringly yet with charity. I, as a fully-trained and experienced nursing sister, agree unhesitatingly with the majority of her opinions, and also with her suggested remedies for many defects—defects which may be too often summed up as crying evils.

"Ierne" lays stress on the fact that nurses are curiously silent on the subjects which so vitally concern them. Among the reasons for this extraordinary dumbness one in particular has not, as far as I am aware, been suggested, yet this reason has far-reaching influences. The majority of nurses trained and in training whose opinions might have weight—I refer more particularly to hospital sisters —do not read the nursing journals, and the initial blame may be laid at the door of that long-suffering woman, "the woman with the whip"! Does she encourage her sisters, staff nurses, and probationers to subscribe to the nursing journals? Does she promote discussion as to new methods of treatment, on nursing news, and on the many interesting topics which are week by week dealt with in the nursing press, and which are bound to affect all nurses in the near or distant future? *No.* That is as far as my personal experience of four large training-schools goes, and, I may add, the personal experience of many nurse friends of varied training-schools. There are, of course, exceptions. Would that there were more!

If every matron of every important and unimportant training-school made a point that the nursing press should freely circulate among the entire staff, not forgetting that important person the impressionable probationer, with a view to the encouragement of free discussion on the many burning topics which continually arise, each matron would be whole-heartedly, or may be half-heartedly, obeyed; but obeyed she would be, *because she wields the whip.*

The result would probably be that nurses would cease to be as inarticulate as they are now, and there might be more professional unity. Matrons do all in their power to cherish the traditions and uphold the prestige of the training-schools they in a measure rule. Then they rest content. Whilst an admirable spirit, it is conducive to a certain narrowness. School may be too often half unconsciously set against school in a none too friendly rivalry. It is undoubtedly pleasant to be brought up in the "my training-school is second to none" atmosphere, but it may evolve an irritating complacency. Hospital schools sneer too often at infirmary schools, the good points of which the hospital schools may never trouble themselves to discover. I cannot resist that last item as one subject at least on which "Ierne" will never find nurses inarticulate —at the risk of wandering from my point.

If "Ierne" would like further proof of my one suggested reason for the silence of nurses I can give more facts. To me it is almost painfully apparent that the power of the nursing press is not only not utilised to the full, but is not even recognised by many influential women—women to whom are entrusted almost unlimited powers of influence and direction.—Yours faithfully,

A Scotch Woman.

